# Supplementary material for: Mechanistic understanding of speciated oxide growth in high entropy alloys
Source: Nat Commun. 2024 Jun 12;15:5026. doi: 10.1038/s41467-024-49243-8 (PMC11169556; doi:10.1038/s41467-024-49243-8)
Supplement: Supplementary file 1 — Supplementary Information [file 41467_2024_49243_MOESM1_ESM.docx]

**Supplementary Information**

**for**

**Mechanistic Understanding of Speciated Oxide Growth in High Entropy Alloys**

Bharat Gwalani^1^*, Andrew Martin^1^, Elizabeth Kautz^2,3^ Boyu Guo^1^, S.V. Lambeets^4^, Matthew Olszta^2^, Anil Krishna Battu^5^, Aniruddha Malakar^1^, Feipeng Yang^6^, Jinghua Guo^6^, Thevuthasan Suntharampillai^4^, Aram Amassian^1^, Ruipeng Li^7^, Martin Thuo^1^, Arun Devaraj^4^*

^1^North Carolina State University, Department of Materials Science and Engineering. Raleigh, NC 27695. USA

^2^Energy and Environment Directorate, Pacific Northwest National Laboratory, Richland, WA 99352 USA

^3^North Carolina State University, Department of Nuclear Engineering. Raleigh, NC 27695. USA

^4^Physical & Computational Sciences Directorate, Pacific Northwest National Laboratory, Richland, WA 99352 USA



^5^Earth and Biological Sciences Directorate, Pacific Northwest National Laboratory, Richland, WA 99352 USA

^6^Advanced Light Source, Lawrence Berkeley National Laboratory, Berkeley, CA, USA

^7^National Synchrotron Light Source II, Brookhaven National Laboratories, Upton, NY, 11973, USA

*Corresponding author emails: bgwalan@ncsu.edu, [arun.devaraj@pnnl.gov](mailto:arun.devaraj@pnnl.gov)

Supplementary Materials



**Table of Contents**

Supplementary Notes 1: Preferential Interactivity Parameter…………………………………………..……… S2

Supplementary Notes 2: APT Sample Preparation and Analysis…………………………………….… ……….S3

Supplementary Notes 3: Solid State Diffusion and Oxide Film Formation……………….………………….S4

Supplementary Notes 4: Circular average of Grazing Incidence Wide Angle X-ray Scattering (GIWAXS) data of oxidized alloy with X-ray traces collected from different incident angles varying from 0.05^o^-0.5^o^ ………………………………………………………………………………………………………………….…….S5

Supplementary Notes 5: APT element distribution maps and contour plots for the CoCrFeNiMi HEA……………………………………………………………………………………………………………………………………….… S7

Supplementary Notes 6: APT element distribution maps, contour plots, and concentration profiles for the CoCrFeNiMn_0.6_Al_0.4_ HEA………………………………………………………………………………….S10

Supplementary Notes 7: Schematic illustration showing the experimental procedure for ex-situ and in-situ characterization of oxide film in the current study ……………………………………………… S13

References……………………………………………………………………………………………………………...……….…… S13

**Supplementary Notes 1: Preferential Interactivity Parameter**

The Preferential Interactivity Parameter (PIP) utilizes elements' intrinsic properties to determine their favorability to either diffuse toward the surface (segregate) or stay within the bulk (mix). Parameters such as atomic radius (*r*, pm), cohesive energy density (*CED*, Pa)^1,2^, and standard reduction potential (*E^0^*, V) are chosen specifically for oxidation processes, whereas atomic radius determines mobility, *CED* determines the strength of interatomic bonds within the bulk hence the energy cost to escape from the native state, and *E^0^* (or electronegativity) determines favorability to oxidize. Convergence of these parameters into a 3D plot, the so-called PIP, predicts preference to dominate the surface in core-shell particles hence oxidation behavior in general.^3^ In general, coupled lower values in each of these parameters results in a preference to diffuse towards the surface in an oxidizing environment, as a small radius means higher mobility, low *CED* (low vapor pressure) indicates weaker bonds hence low energy cost to leave the native state and lower diffusion energy barrier *en route.* Low *E^0^* gives the propensity of an element to oxidize (i.e. lose electrons) hence a higher chemical potential towards an oxidizing front. The role of radius is captured by the Stokes-Einstein-Sutherland equation (based on charge density) whereas diffusion is inversely proportional to the element’s size ($D\propto\frac{1}{r}$). Differences in size also affect favorability in the ability of a certain element to fill interstitial gaps during oxidation.

Shifting from thermodynamics (prediction) to kinetics, in a multi-element system, a gradient in diffusion towards the surface is expected based on species ranking on the PIP. Surface speciation has previously been demonstrated in metal alloys through spectroscopy, theory, or composition inversion.^4-8^ Thermodynamically, speciation within the surface oxide (Guggenheim interface) is critical in developing pseudo-equilibrium conditions between a system and its surroundings as it serves as an energy and mass interface barrier. Consequently, the system needs to accommodate the associated energy gradient due to Coulombic effects (e.g. dipoles) associated with compositional gradients. PIP helps predict which elements are more favorable to surface segregation and the likely distribution in the sub-oxide layers and/or interface-enriched components between the oxide and the bulk alloy. The combination of this energetic and composition gradient is the building block for the surface tensor that is developed within the surface oxide, $<\hat{T}>=\nabla\Gamma_{i}\mu_{i}$ (where $\Gamma$ is interfacial excess and $\mu$ is chemical potential).

**Supplementary Notes 2: Sample Preparation for Atom Probe Tomography (APT) and Analysis**

APT samples were made via the focused ion beam (FIB) lift-out procedure, using a dual beam focused ion beam-scanning electron microscope (FIB-SEM). Final APT specimens (an example of which is shown in Figure S1(a)) were shaped into needles with an apex diameter of < 50 nm using 2 kV Ga to minimize Ga ion beam damage and implantation. For in situ experiments, a pre-sharpened tungsten TEM-APT grid,^9^ shown in Figure S1(b) was used.

**
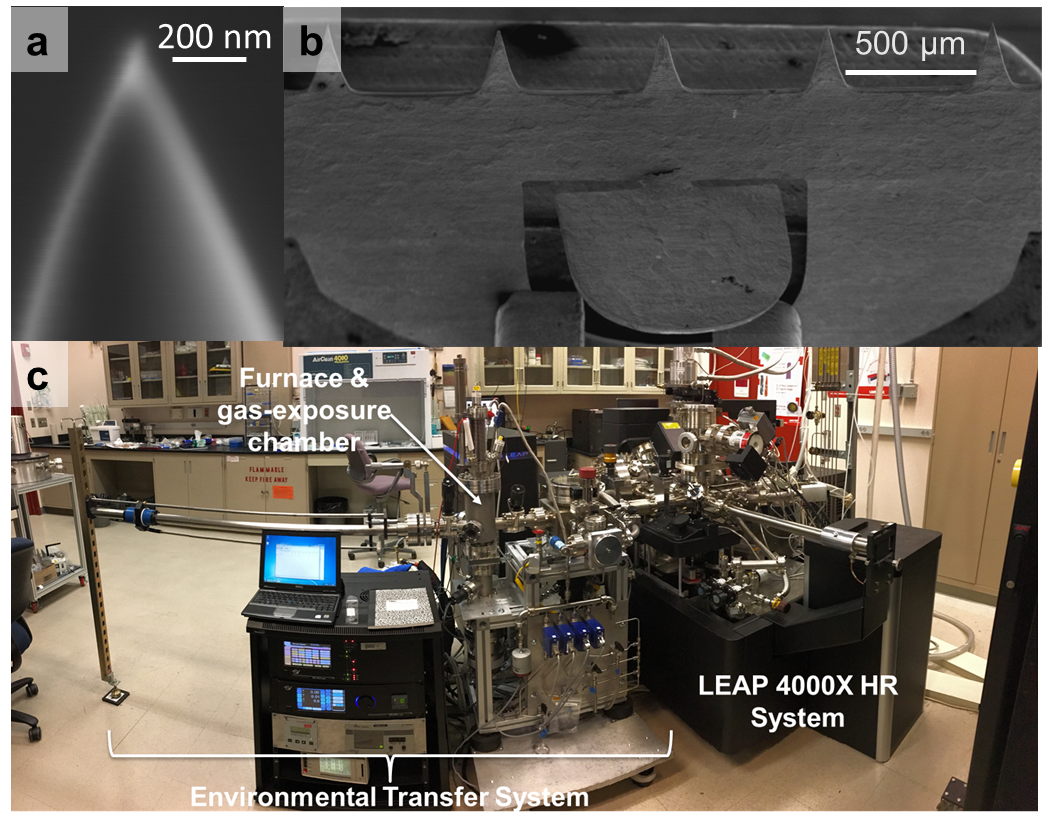
**The APT system used for in situ experiments and data collection is shown in Figure S1(c). A CAMECA local electrode atom probe (LEAP) 4000X HR system equipped with a 355 nm wavelength ultraviolet (UV) laser was used for APT data collection with the following analysis parameters: 60–200 pJ/pulse laser energy, 125 kHz pulse repetition rate, 45 K specimen base temperature, and 0.003 detected ions/pulse detection rate. The analysis chamber pressure was kept at less than 2×10^-11^ Torr. The detector efficiency of the LEAP used in this work is approximately 36%. The custom-built environmental transfer system was used for all oxygen gas exposures.

**Figure S1.** The needle shaped specimens were deposited on a TEM-APT custom-made conical TEM grid. An image of the polished APT need and the grid is provided in (a) and (b) respectively. (c) The state-of-the-art customized APT instrument which has a reaction chamber attached to the atom probe. More details on the modified equipment can be found in the literature by Lambeets, et. al.^10^

**Suppmentary Notes 3: Solid-State Diffusion and Formation of Oxide Film**

Figure S2 displays significant dislocation and cracks within the interface between the oxide and base alloy (enriched zone) due to the oxidation occurring exclusively within the solid state of this alloy (400°C). As captured by the Shuttleworth equation,^11^ unlike oxidation in the liquid state—where surface atoms can plastically deform and reorganize, solid-state oxidation induces elastic deformation causing surface defects, especially within the enriched zone. Another consequence of solid-state diffusion leads to speciation and the creation of defined oxide zones such as the visible Cr_2_O_3_ *in lieu* of more diffuse (statistical) layers (Figure 2c). The defects that are created lead to a new diffusion pathway for metal atoms toward the surface, this can be seen from the formation of metallic lattice towards the surface (hypothetically diffused Co as shown from STEM-EDS and APT results) above the developed Cr_2_O_3_ and spinel.

**
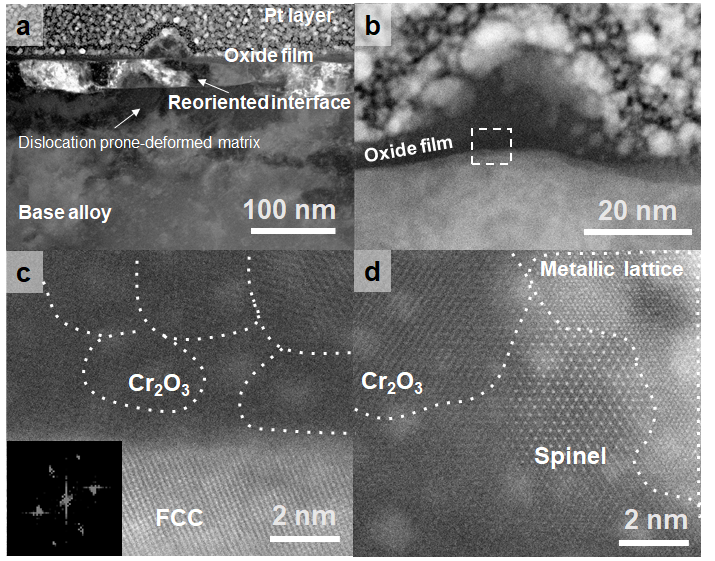
**

**Figure S2.** TEM results from the oxidized CoCrFeNiMn HEA (120 mins). The early stage of oxide film is complex, multiphase and nanocrystalline (a) A TEM BF image showing the cross-section of the alloy where the base, enriched interface (brightened layer) and oxide film can be seen. The top layer is Pt deposited during the FIB-liftout process. (b) A closer look at the oxide-alloy interface, the swelling, and bubble-like formation depict oozing out of Fe/Co/Mn metal/ions from the Cr-oxide layer to form the top-layer oxides. (c) A higher magnification view of the FCC (HEA) and Cr_2_O_3_ interface shows the nano-crystalline nature of the chromia layer. (d) A region magnified from the interface of Cr_2_O_3_ and the topmost multi-oxide (Fe/Co/Mn) region. This region shows the presence of chromia with a corundum structure, a spinel structure and a Co-rich metallic lattice structure indicating pure Co diffusing outwards.

**Supplementary Notes 4: Circular average of Grazing Incidence Wide Angle X-ray Scattering (GIWAXS) data of oxidized alloy with X-ray traces collected from different incident angles varying from 0.05^o^-0.5^o^**


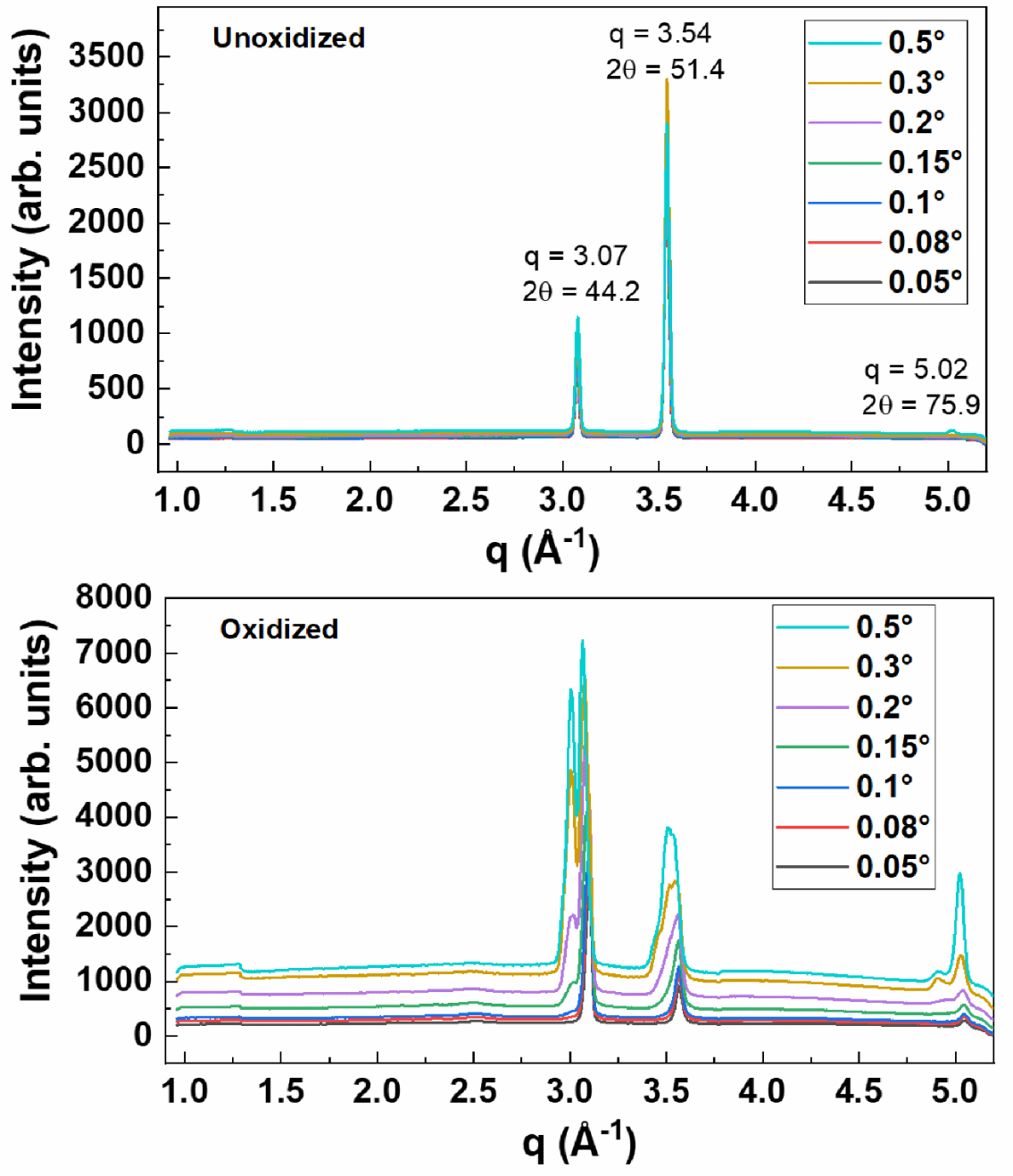


**Figure S3:** Circular average of GIWAXS comparing the unoxidized and oxidized samples.

**
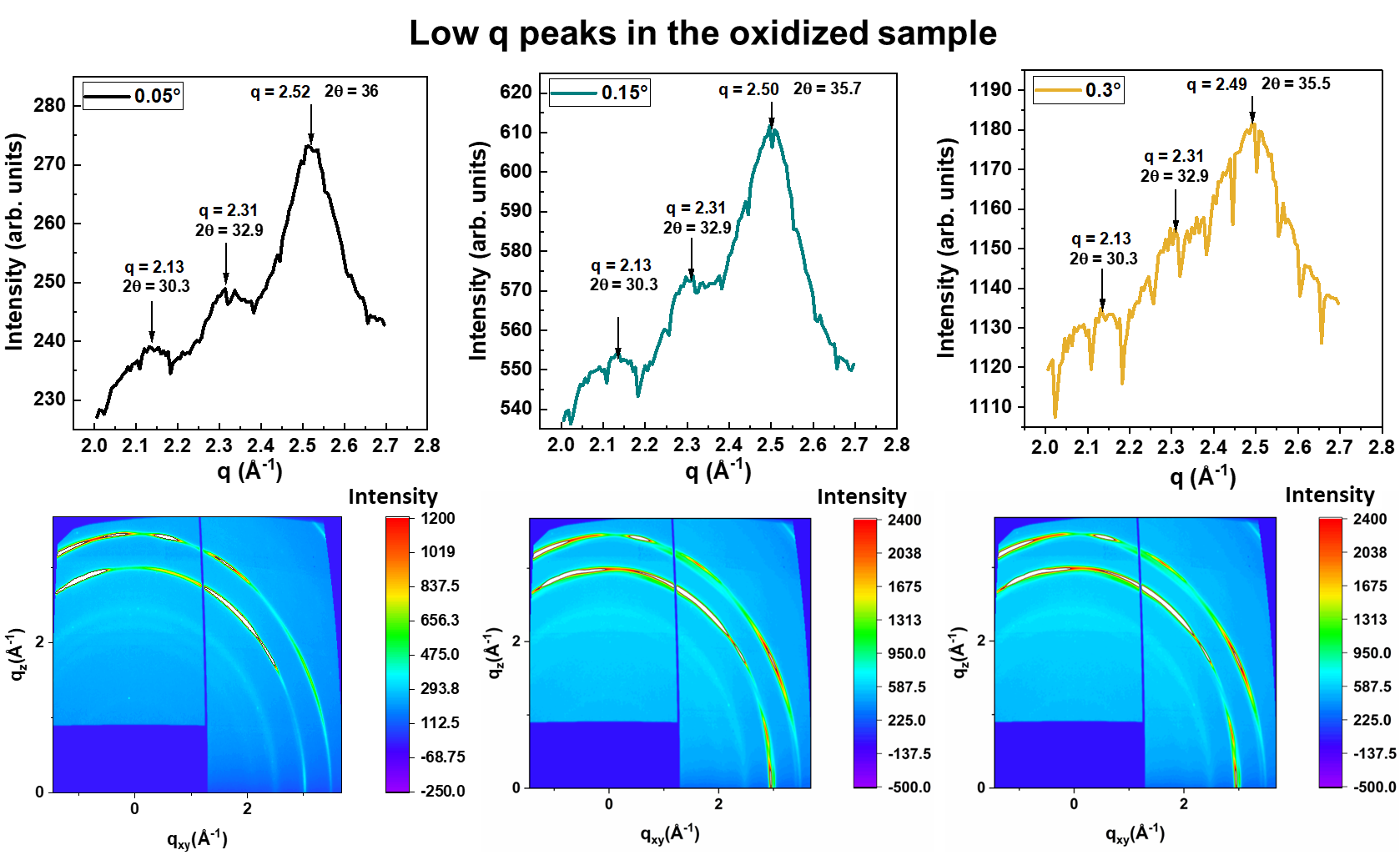
**

**Figure S4:** Snapshots showing the change in the low q peaks in oxidized sample as the incident angle is changes from low (0.05^o^) to high (0.3 ^o^)

**Supplementary Notes 5: APT element distribution maps and contour plots for the CoCrFeNiMn HEA**

Three-dimensional (3D) element distribution maps and two-dimensional (2D) contour plots for APT reconstructed volumes for 2-minute, 30-minute, and 2-hour oxidation exposure are provided in Figures ~~S4 - S6~~ S5-S7. The 3D element distribution maps all show a 10 nm section from the center of the reconstructed volume to easily visualize the oxide/metal interfaces. Iso-concentration surfaces in both 3D element distribution maps and 2D contour plots provide an estimate of the location of the oxide/metal interfaces. This iso-concentration surface composition varies with oxidation exposure time and varies from 20 - 35 at. % oxygen (O).

**
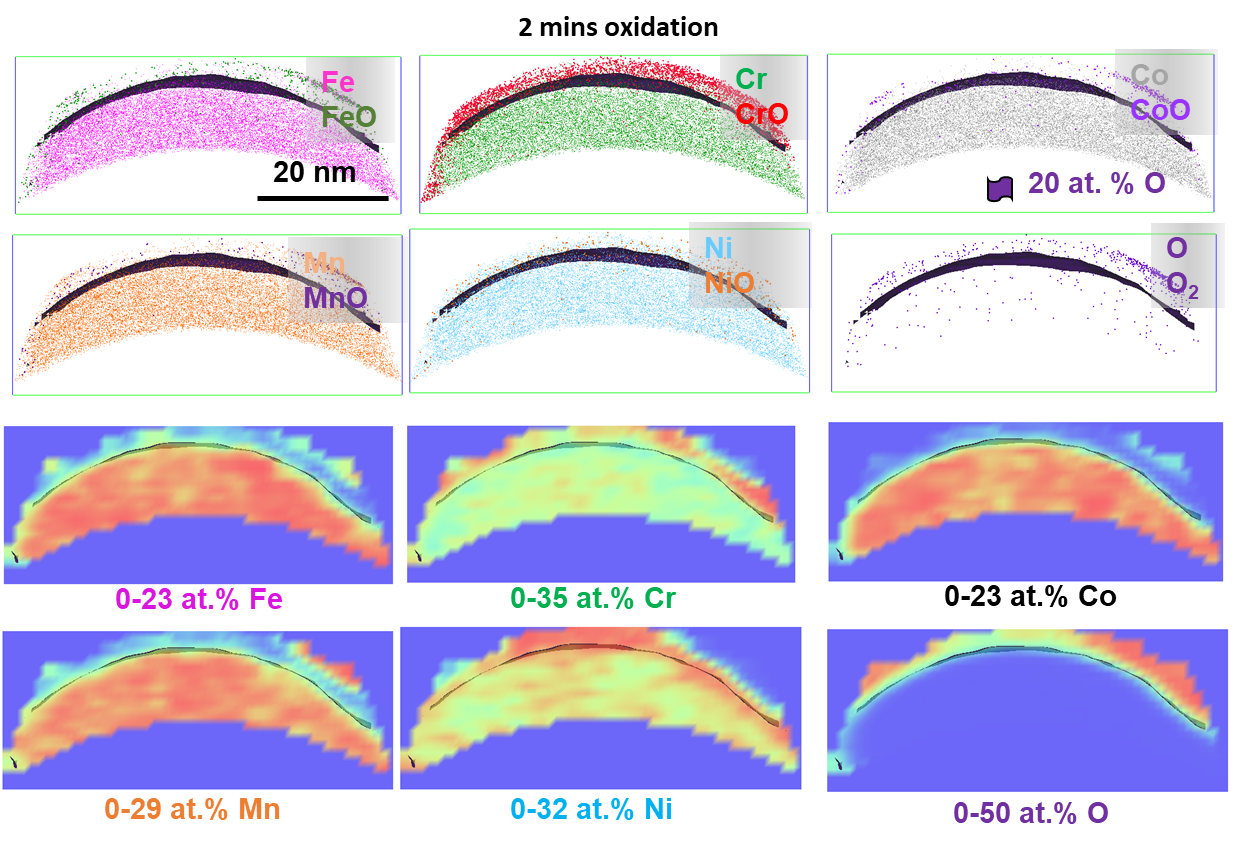
**

**Figure S5.** APT results from the 2 mins oxidation treatment. In the top panel, the ion maps of Fe/FeO, Cr/CrO, Co/CoO, Mn/MnO, Ni/NiO and O/O_2_ show the distribution of pure metal and oxides species of that metal in each image. Below them are the thermal maps showing the density of each in the 2D map.

**
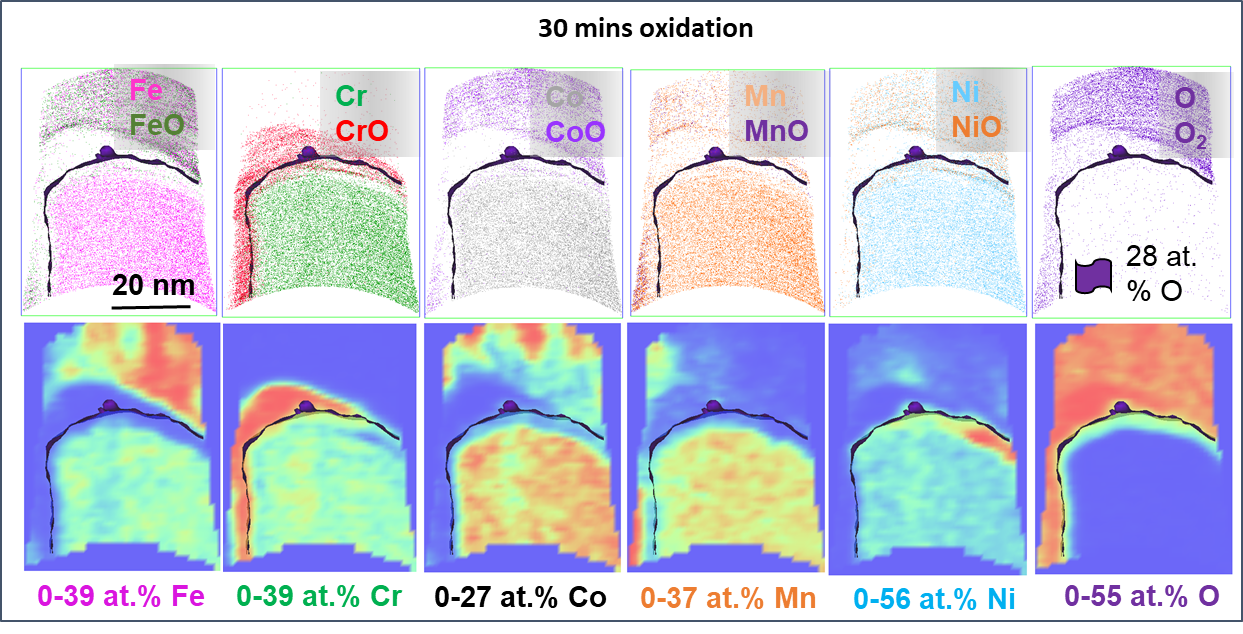
Figure S6.** APT results from the 30 mins oxidation treatment. In the top panel, the ions maps of Fe/FeO, Cr/CrO, Co/CoO, Mn/MnO, Ni/NiO and O/O_2_ show the distribution of pure metal and oxides species of that metal in each image. Below them are the thermal maps showing the density of each in the 2-D map.

**
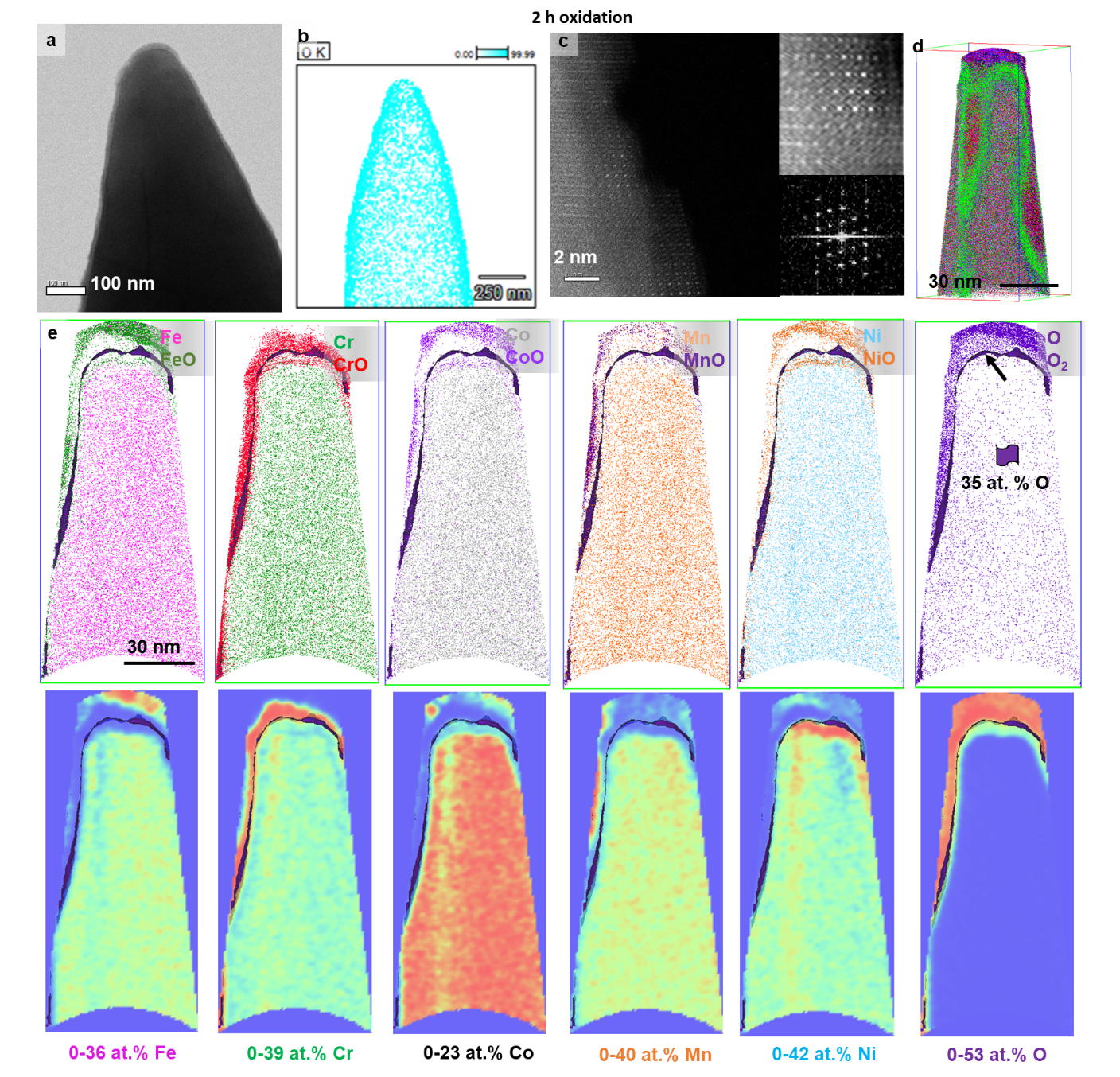
Figure S7.** (a-c) have the TEM results showing the needle after 120 mins of oxidation. The needle was transported from the APT chamber to TEM under a controlled environment. The high-resolution imaging of the needle was not possible at most locations due to the high thickness of the needle, however, at few locations the atomic structure of the out region was captured using atomic column STEM images as seen in figure (c). The region captured in (c) depicted a corundum structure indicating a chromia layer.

**Supplementary Notes 6: APT element distribution maps, contour plots, and concentration profiles for the CoCrFeNiMn_0.6_Al_0.4_ HEA**

**
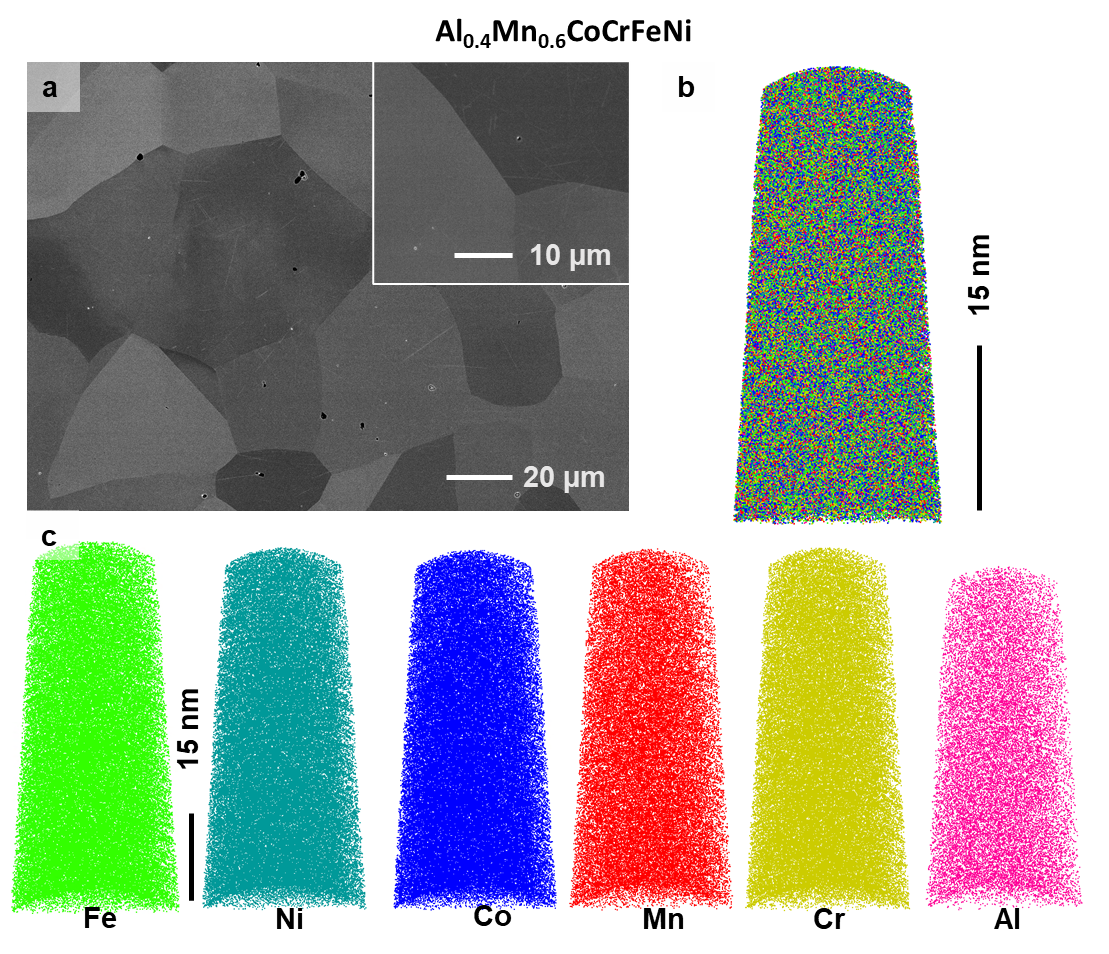
**

**Figure S8.** SEM and APT results from the modified alloy where Al substituted Mn partially. The melting, cold rolling (50%), annealing at 1100 C for 30 mins and water quenching resulted in a recrystallized equiatomic grain structure as seen in Figure (a). The APT structure in (b-c) show a single phase random alloy no perceivable compositional partitioning.

**
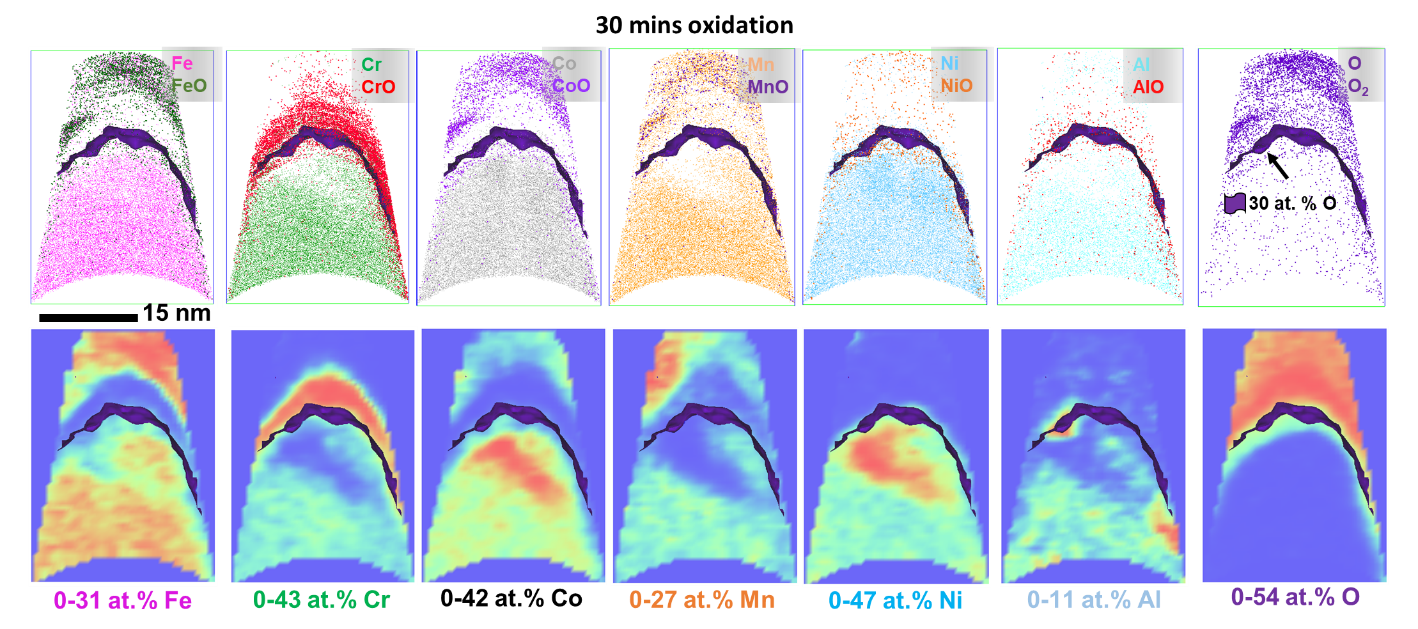
Figure S9.** APT results from the 30 mins oxidation treatment of AlMnCoCrFeNi alloy. In the top panel the ions maps of Fe/FeO, Cr/CrO, Co/CoO, Mn/MnO, Ni/NiO, and O/O2 show the distribution of pure metal and oxides species of that metal in each image. Below them are the thermal maps showing the density of each in the 2-D map.

From the 3D element distribution maps and 2D contour plots shown in Figure ~~S8~~ S9, we see that the inner oxide is Cr-rich, and the outer oxide includes Fe, Co, and Mn signals. To further investigate the composition of the outer oxide, concentration profiles were generated across Fe and Mn iso-concentration surfaces, with results reported in Figure S10, from the concentration profile across the 33 at. % iso-concentration surface, we see that the Fe-rich regions of the outer oxide also have approximately 10 at. % Co, but other elements, including Mn, Cr, Ni, and Al are less than a few at. % (Figure S10(b)). We also find there is a pile-up of Mn at the outer region of the Fe-rich outer oxide (up to approximately 10 at. % Mn). In the Mn-rich region of the outer oxide (shown in Figure S10(c)-(d)), we also observe signals from Fe, Co, and Ni. From Figure ~~S9~~ S10 we see that the outer oxide of the CoCrFeNiMn_0.6_Al_0.4_ HEA has non-uniform composition, with Fe and Mn-enriched regions, suggesting non-uniform mobility of these elements through the Cr-rich inner oxide.


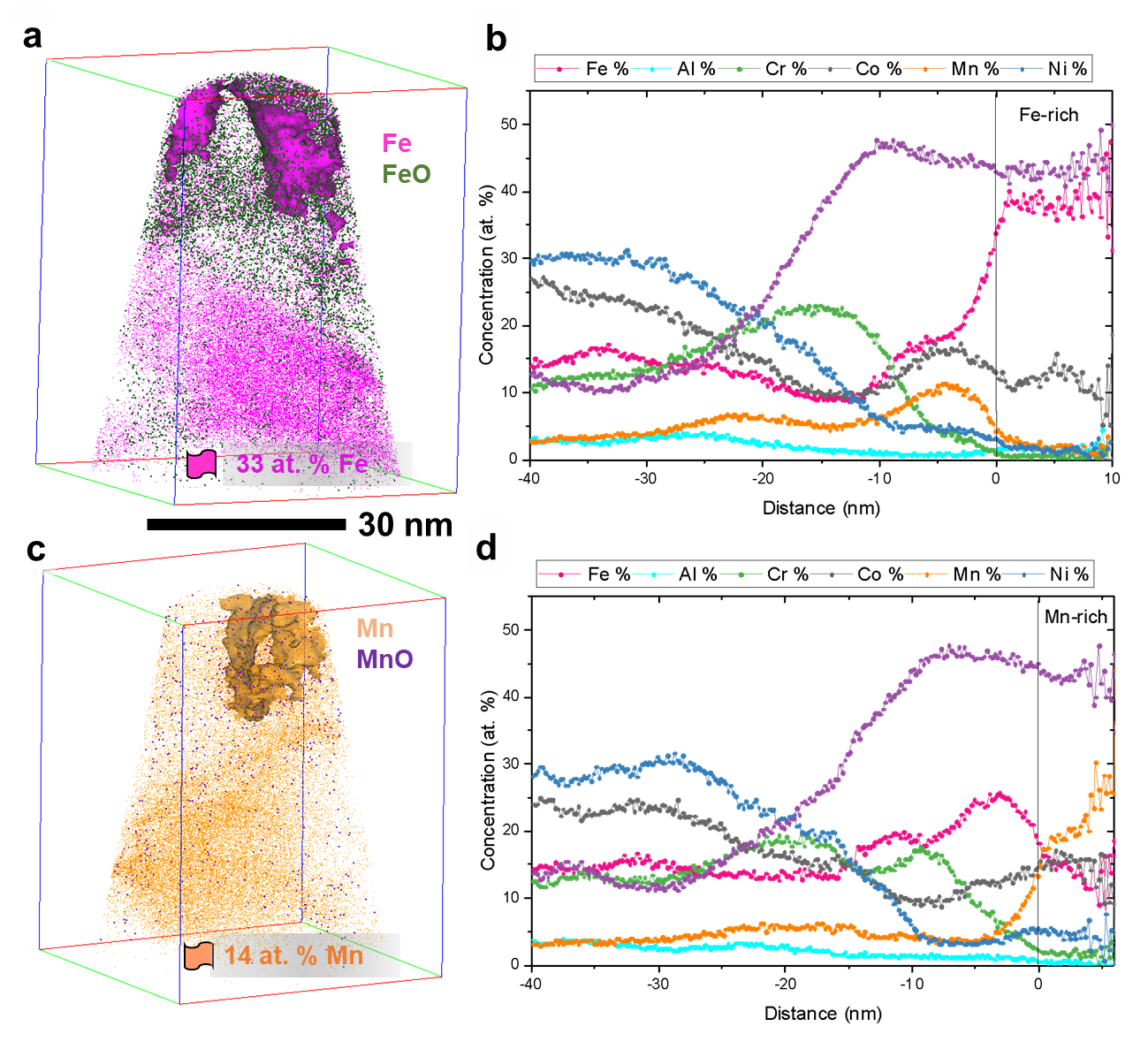


**Figure S10.** APT results from the 30 mins oxidation treatment of AlMnCoCrFeNi alloy. (a) shows the Fe and FeO ions to depict the oxide layer. (b) shows the concentration profile across the 33 at. % Fe iso-concentration surface. (c) Mn rich region at the outer oxide. (d) shows the concentration profile across the 14 at. % Mn iso-concentration surface.

**Supplementary Notes 7: Schematic illustration showing the experimental procedure for ex-situ and in-situ characterization of oxide film in the current study**


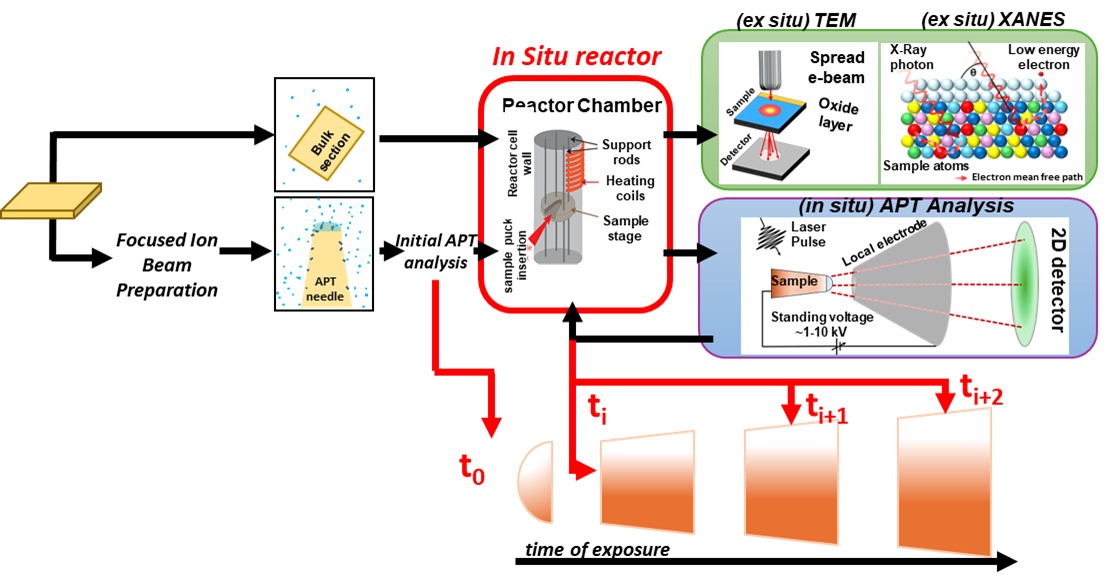


**Figure S11.** A schematic of experimental procedure for coupled ex-situ and in-situ characterization of oxide film formation on HEA surfaces: The process involves the oxidation of a bulk sample in the form of a flat specimen for ex-situ TEM and XANES analyses, as well as preparing APT needles for in situ investigations. All oxidation experiments were conducted within the same chemical reactor chamber environment.





**Figure S12.** Schematic illustration of the method employed for performing ex-situ GIWAXS experiments to evaluate the crystal structures of oxide layers on a bulk sample.

**Supplementary References**

1 Wang, L.-L. & Johnson, D. D. Predicted Trends of Core−Shell Preferences for 132 Late Transition-Metal Binary-Alloy Nanoparticles. *J. Am. Chem. Soc.* **131**, 14023-14029 (2009).

2 Mizutani, U., Inukai, M., Sato, H. & Zijlstra, E. S. in *Physical Metallurgy (Fifth Edition)* (eds David E. Laughlin & Kazuhiro Hono) 103-202 (Elsevier, 2014).

3 Martin, A., Chang, B. S. & Thuo, M. Effect of Surface Nanostructures and Speciation on Undercooling for Low-Temperature Solder Alloys. *ACS Appl. Nano Mater.* **5**, 3325-3332 (2022).

4 Cutinho, J. *et al.* Autonomous Thermal-Oxidative Composition Inversion and Texture Tuning of Liquid Metal Surfaces. *ACS Nano* **12**, 4744-4753 (2018).

5 Martin, A. *et al.* Passivation-driven speciation, dealloying and purification. *Mater. Horiz.* **8**, 925-931 (2021).

6 Martin, A., Kiarie, W., Chang, B. & Thuo, M. Chameleon Metals: Autonomous Nano-Texturing and Composition Inversion on Liquid Metals Surfaces. *Angew. Chem. Int. Ed.* **59**, 352-357 (2020).

7 Cademartiri, L. *et al.* Electrical Resistance of Ag^TS^–S(CH_2_)_n−1_CH_3_//Ga_2_O_3_/EGaIn Tunneling Junctions. *J. Phys. Chem. C* **116**, 10848-10860 (2012).

8 Sodhi, R. N. S., Brodersen, P., Cademartiri, L., Thuo, M. M. & Nijhuis, C. A. Surface and buried interface layer studies on challenging structures as studied by ARXPS. *Surf. Interface Anal.* **49**, 1309-1315 (2017).

9 Felfer, P. J., Alam, T., Ringer, S. P. & Cairney, J. M. A reproducible method for damage-free site-specific preparation of atom probe tips from interfaces. *Microsc. Res. Tech.* **75**, 484-491 (2012).

10 Lambeets, S. V. *et al.* Nanoscale Perspectives of Metal Degradation via In Situ Atom Probe Tomography. *Top. Catal.* **63**, 1606-1622 (2020).

11 Shuttleworth, R. The Surface Tension of Solids. *Proc. Phys. Soc. A* **63**, 444 (1950).
